# Morphological study of the popliteus muscle-tendon complex in formalin embalmed adult cadavers

**DOI:** 10.12688/f1000research.141366.2

**Published:** 2023-12-21

**Authors:** Rajanigandha Vadgaonkar, Mamatha Tonse, Vandana Blossom, P. Gopal Govind Kalluraya, B.V. Murlimanju

**Affiliations:** 1 Department of Anatomy, Kasturba Medical College, Mangalore, Manipal Academy of Higher Education, Manipal, Karnataka, India

**Keywords:** Arthroscopic Surgery; Popliteus; Plastic Surgery

## Abstract

**Background:**

The dimensions of popliteus muscle and its tendon are subjected to variability in the origin, mode of insertion, innervation patterns and vascular supply. The aim of this study was to measure the length, thickness and width of the popliteus muscle and its tendon at its different parts. The objectives were to study the topographic anatomy of the neurovascular structures of the popliteus and also to determine the dimensions of the popliteo-fibular ligament.

**Methods:**

This descriptive cross sectional institutional based study included 50 formalin embalmed adult lower limb specimens. The measurements were performed by using the digital Vernier caliper.

**Results:**

The length of the popliteus muscle belly along the upper and lower border were 44.2±6.63 mm and 89.26±14.41 mm, width of the muscle belly at midpoint, musculotendinous junction and insertion were 28.45±6.85 mm, 11.7±3.5 mm and 75.95±10.7 mm. The thickness of muscle belly at the midpoint was 2.55±0.55 mm. The length of popliteal tendon, width at origin and at musculotendinous junction were 24.85±2.15 mm, 7.55±1.55 mm and 8.5±1.15 mm. The thickness of tendon of popliteus was 2.6±0.75 mm. The length of nerve to popliteus was 50.44±8.66 mm and its origin was located 27.54±6.18 mm from the intercondylar line. The distance of origin of medial and lateral geniculate arteries from the intercondylar line were 26.26±10.47 mm and 20.76±5.19 mm. The distance of division of popliteal artery was 49.44±16.26 mm from the intercondylar line. The length and width of the popliteo-fibular ligament was 17.84±3.43 mm and 7.36±1.9 mm individually.

**Conclusions:**

This study offered detailed morphometric data of the popliteus and it is believed that the data of this anatomical research is enlightening to orthopedic surgeons particularly in the field of arthroscopic and plastic surgery. The data can be considered as the database from our population.

## Introduction

The popliteus is a muscle in the posterior compartment of the lower extremity, which is located at the leg and innervated by the tibial nerve. This is the only muscle in the back of leg, which acts on the knee joint and not over the ankle joint. This is considered as the unlocking muscle of the knee joint. ^1^ It laterally rotates the femur over the tibia, while walking when one foot is on the ground. It helps in the knee stabilization along with the fibular collateral and popliteo-fibular ligaments. ^1^ The popliteus has dual origin, one from the lateral femoral condyle and the other from the lateral meniscus. Its origin is tendinous and it is interesting to know that there exists variability in its origin like from the styloid process of the fibula. ^2^ On few occasions, an accessory head of popliteus may originate from the sesamoid bone at the gastrocnemius lateral head. ^3^ On rare occasions, there may be a popliteus minor muscle, which originates from the femur over the deep part of the plantaris muscle and its distal attachment is at the posterior aspect of knee joint. ^2^ The popliteal tendon occupies a part of the knee joint capsule; however, it does not enter the synovial cavity. Hence it is intra-capsular, however extra-articular and extra-synovial. ^4^ It runs underneath the fibular collateral ligament and biceps femoris tendon. The popliteus separates the lateral collateral ligament from the lateral meniscus and prevents its injury. ^5^ The popliteus inserts at the dorsal aspect of the tibial upper end just over the soleal line. More clinical and basic anatomical studies are needed to understand the injuries and pathological involvement of popliteus in order to accomplish the better diagnosis and management. ^6^ In this situation, the primary goal was to determine the dimensions of different parameters of popliteal muscle tendon complex at its various parts. The objectives were to measure the dimensions of popliteo-fibular ligament and to study the topographic anatomy of the neurovascular structures supplying the popliteus.

## Methods

**Figure 1.**
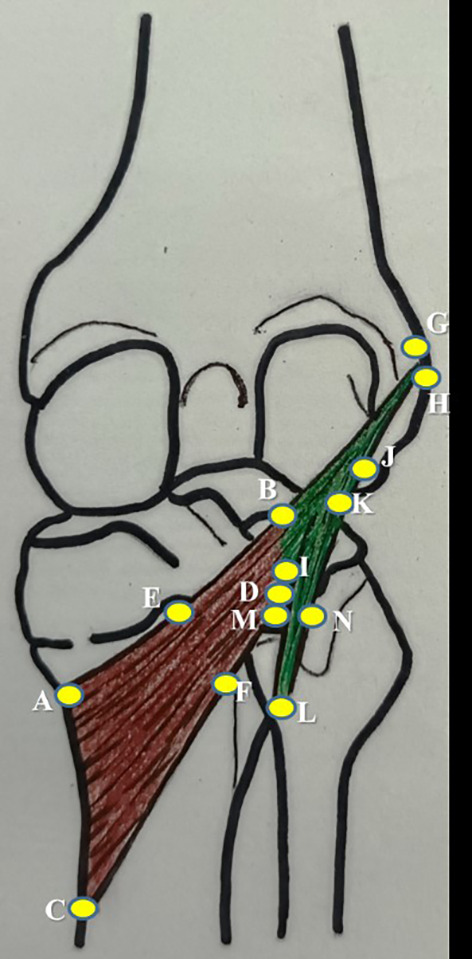
Schematic representation of the measurements of popliteus muscle tendon complex, which are performed in this study (refer to the details in Table 1).

This is a descriptive cross sectional institutional based study, which involved 25 formalin embalmed adult cadavers from Indian population. The sample size is similar to the earlier study performed by Olewnik *et al*. ^1^ The protocol of this anatomical research is available at dx.doi.org/10.17504/protocols.io.3byl4qqk8vo5/v1. Meticulous dissection was performed to expose the popliteus muscle, tendon and its neurovascular structures. In total, 50 popliteus muscles were analyzed based on the side. The sex differences were not explored in this study. The inclusion criteria were adult embalmed cadavers, which were available at the department of anatomy. Cadavers showing pathological changes and congenital anomalies at the knee joint were excluded from this study. The exclusion criteria also included the previously dissected cadavers. The measurements were performed by using the digital Vernier caliper (Mitutoyo Digital Vernier Caliper 0-150 mm 500-196 made in Japan) and the analysis of the data was done by using the recent version of SPSS (version 27) software after applying the paired t-test. Single person, who is a coauthor in this study, performed all the measurements. This was followed to prevent the inter-observer bias and the measurements were taken on three consecutive times. The average of which was considered to prevent the intra-observer bias. The measurements of the popliteus muscle tendon complex are schematically represented in Figure 1 and tabulated in Table 1.

**Table 1.** Measurements of the popliteus muscle tendon complex performed in this study.

No.	Parameter	Representation in Figure 1
1	mediolateral length along the upper border of popliteus	AB
2	mediolateral length along the lower border of popliteus	CD
3	width at the muculotendinous junction of popliteus	BD
4	width at the midpoint of popliteus	EF
5	width at the insertion of popliteus	AC
6	thickness at the midpoint of popliteus along the lower border of popliteus	F
7	length of popliteal tendon	BG
8	width of popliteal tendon at origin	GH
9	width of politeal tendon at muculotendinous junction	BI
10	thickness of popliteal tendon at mid point	J
11	distance of origin of medial geniculate artery from the intercondylar line	-
12	distance of origin of lateral geniculate artery from the intercondylar line	-
13	distance of division of popliteal artery from the intercondylar line	-
14	distance of origin of nerve to popliteus from the intercondylar line	-
15	length of nerve to popliteus	-
16	length of popliteo-fibular ligament	KL
17	width of popliteo-fibular ligament	MN

This anatomical research has received the approval from the ethics committee of our institution (Approval Committee Name: Institutional Ethics Committee, Kasturba Medical College, Mangalore, Approval Number: IEC KMC MLR: 09/2022/400, dated 21.09.2022). Since this is a study from the human cadavers, the consent from the participants is not applicable. This was waived by our institutional ethics committee. The consent was already given by the participant to perform the medical teaching and research, while donating his or her body. This present research is following the guidelines of the international ethical standards.

## Results

The length of the popliteus muscle belly along the upper and lower border were 44.2±6.63 mm and 89.26±14.41 mm, width of the muscle belly at midpoint, musculotendinous junction and insertion were 28.45±6.85 mm, 11.7±3.5 mm and 75.95±10.7 mm. ^18^ The thickness of muscle belly at the midpoint was 2.55±0.55 mm. The morphometric data of the popliteus muscle belly are given in Table 2. The length of popliteal tendon, width at origin and at musculotendinous junction were 24.85±2.15 mm, 7.55±1.55 mm and 8.5±1.15 mm. The thickness of tendon of popliteus was 2.6±0.75 mm. Table 3 represents the dimensions of the tendon of popliteus of this study.

**Table 2.** Morphometric data of the popliteus muscle belly (n=50).

Dimension of the muscle	mean±SD
mediolateral length along upper border	44.2±6.63
mediolateral length along lower border	89.26±14.41
width at the musculotendinous junction	11.7±3.5
width at the midpoint	28.45±6.85
width at the insertion	75.95±10.7
thickness at the midpoint along lower border	2.55±0.55

Measurements are in mm.

**Table 3.** Morphometric data of the tendon of popliteus (n=50).

Dimension of the tendon	mean±SD
length	24.85±2.15
width at origin	7.55±1.55
width at musculotendinous junction	8.5±1.15
thickness	2.6±0.75

Measurements are in mm.

The length of nerve to popliteus was 50.44±8.66 mm and its origin was located 27.54±6.18 mm from the intercondylar line. The distance of origin of medial and lateral geniculate arteries from the intercondylar line were 26.26±10.47 mm and 20.76±5.19 mm. The distance of division of popliteal artery was 49.44±16.26 mm from the intercondylar line. Table 4 offers the topographic anatomy of the neurovascular structures of popliteus. The length and width of the popliteo-fibular ligament was 17.84±3.43 mm and 7.36±1.9 mm individually. They are summarized in Table 5 and the sidewise comparison of all the parameters, which are measured in this study are given in Table 6, Table 7, Table 8 and Table 9. The statistical significance was not there, when the right and left side comparison was considered (p>0.05). The only significant difference was observed for the width of the popliteal muscle at the insertion, which was higher for the left side (p<0.05).

**Table 4.** Topography of the neurovascular structures of popliteus (n=50).

Neurovascular supply to popliteus	mean±SD
distance of origin of MGA from the ICL	26.26±10.47
distance of origin of LGA from the ICL	20.76±5.19
distance of division of PA from the ICL	49.44±16.26
distance of origin of NP from the ICL	27.54±6.18
length of NP	50.44±8.66

Measurements are in mm, MGA - medial geniculate artery; LGA - lateral geniculate artery; ICL - intercondylar line; PA - popliteal artery; NP - nerve to popliteus.

**Table 5.** Morphometric data of the popliteo-fibular ligament (n=50).

Popliteo-fibular ligament	mean±SD
length	17.84±3.43
width	7.36±1.9

Measurements are in mm.

**Table 6.** Sidewise comparison of morphometric data of the popliteus muscle belly (n=50).

Dimension of the muscle	Right side (n=25)	Left side (n=25)
mediolateral length along upper border	44.88±7.44	43.52±5.82
mediolateral length along lower border	88.12±17.06	90.4±11.77
width at the musculotendinous junction	12.4±4.3	11±2.7
width at the midpoint	28.8±6.5	28.1±7.2
width at the insertion *	72.5±9.9	79.4±11.5
thickness at the midpoint	2.6±0.7	2.5±0.4

Measurements are in mm, mean±standard deviation, no statistical significant difference (p>0.05) except.

* (p<0.05).

**Table 7.** Sidewise comparison of morphometric data of the tendon of popliteus (n=50).

Dimension of the tendon	Right side (n=25)	Left side (n=25)
length	25±2.5	24.7±1.8
width at origin	7.1±1.8	8±1.3
width at musculotendinous junction	8.6±1.2	8.4±1.1
thickness	2.4±0.7	2.8±0.8

Measurements are in mm, mean±standard deviation, no statistical significant difference (p>0.05).

**Table 8.** Sidewise comparison of the topography of the neurovascular structures of popliteus (n=50).

Neurovascular supply to popliteus	Right side (n=25)	Left side (n=25)
distance of origin of MGA from the ICL	23.24±9.54	29.28±11.41
distance of origin of LGA from the ICL	20.72±4.96	20.8±5.42
distance of division of PA from the ICL	46.76±18.61	52.12±13.92
distance of origin of NP from the ICL	28.56±7.32	26.52±5.04
length of NP	51.4±8.44	49.48±8.89

Measurements are in mm, mean±standard deviation, no statistical significant difference (p>0.05); MGA - medial geniculate artery; LGA - lateral geniculate artery; ICL - intercondylar line; PA - popliteal artery; NP - nerve to popliteus.

**Table 9.** Sidewise comparison of the morphometric data of the popliteo-fibular ligament (n=50).

Popliteo-fibular ligament	Right side (n=25)	Left side (n=25)
length	17.84±3.23	17.84±3.63
width	6.92±1.57	7.8±2.23

Measurements are in mm, mean±standard deviation, no statistical significant difference (p>0.05).

**Figure 2.**
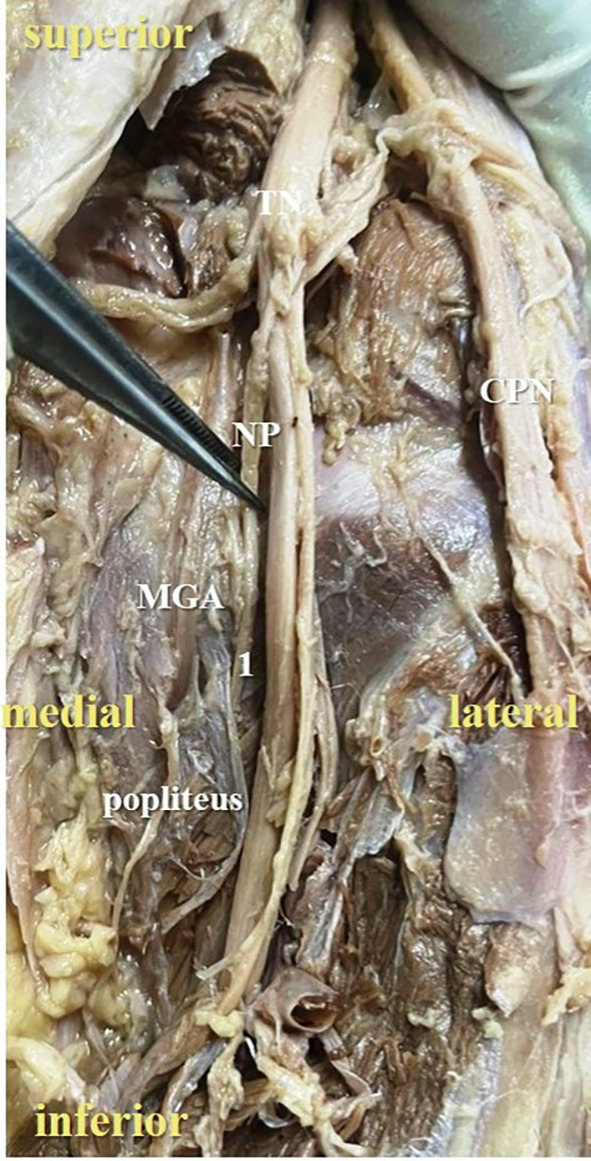
Posterior view of the right lower limb of the cadaver showing the nerve to popliteus (NP), which was giving single twig (34% cases) to popliteus (1-single twig; TN-tibial nerve; CPN-common peroneal nerve; MGA-middle genicular artery).

**Figure 3.**
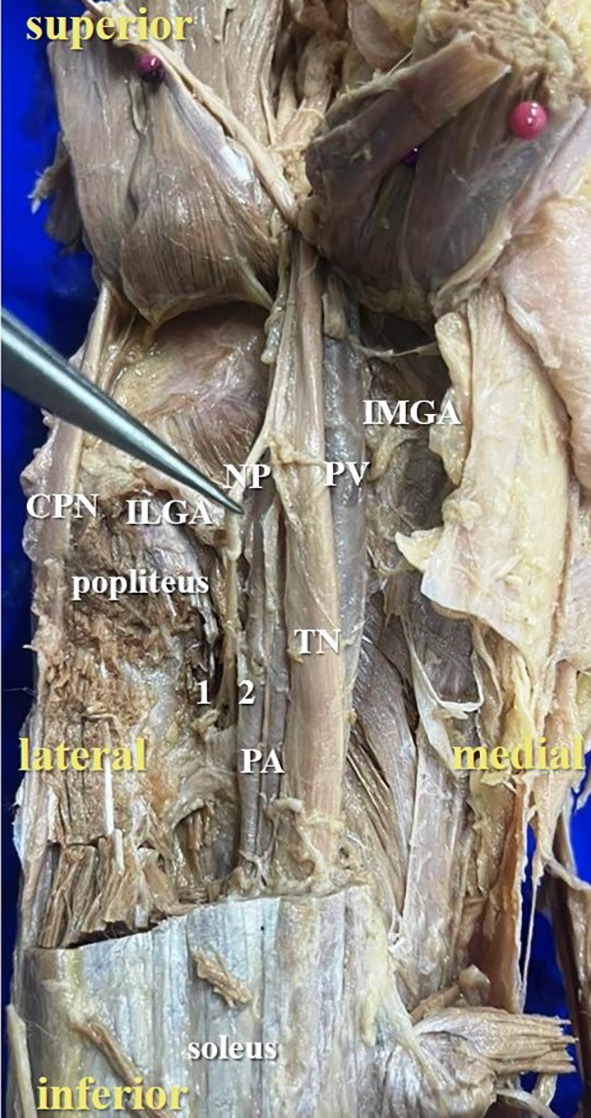
Posterior view of the left lower limb of the cadaver showing the nerve to popliteus (NP), which was giving double twig (42% cases) to popliteus (1-first twig; 2-second twig; TN-tibial nerve; CPN-common peroneal nerve; IMGA-inferior medial genicular artery; ILGA-inferior lateral genicular artery; PA-popliteal artery; PV-popliteal vein).

**Figure 4.**
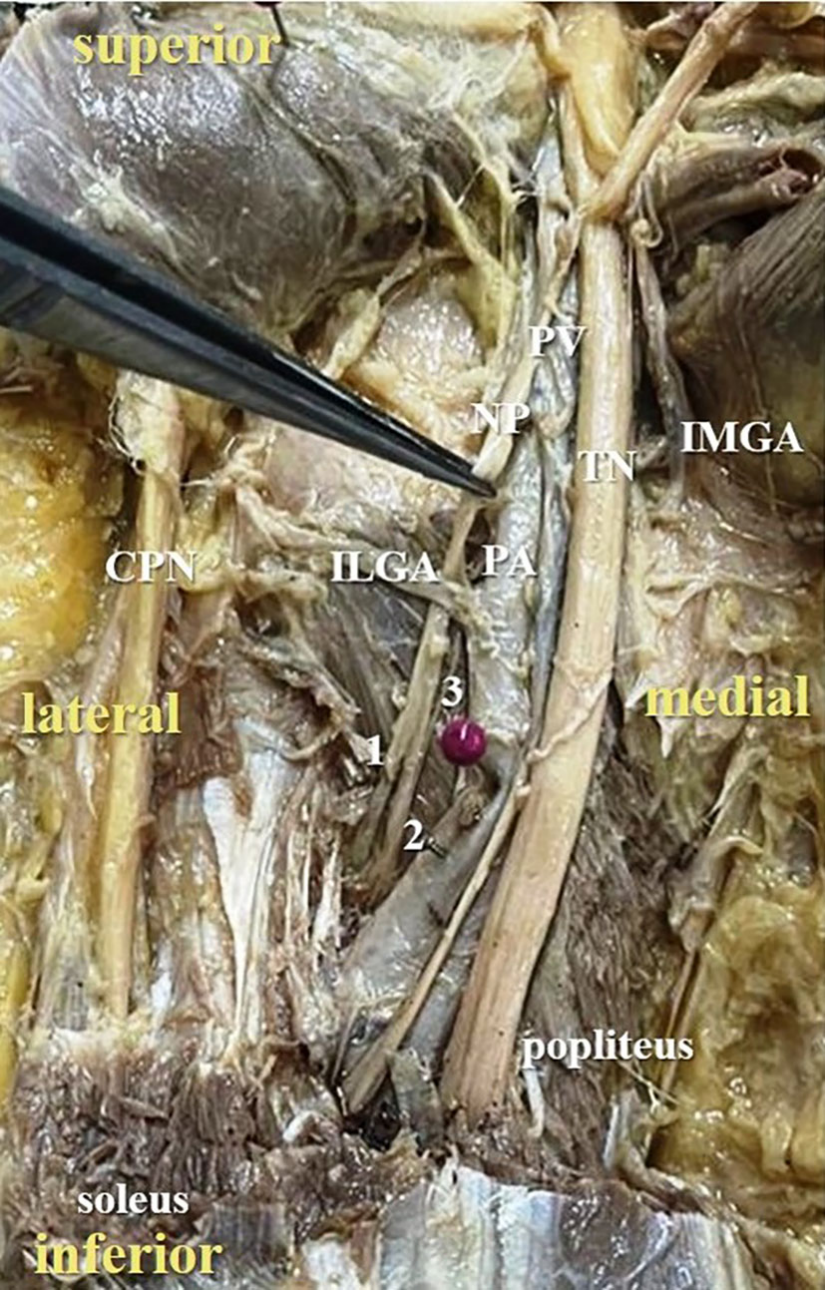
Posterior view of the left lower limb of the cadaver showing the nerve to popliteus (NP), which was giving three twigs (24% cases) to popliteus (1-first twig; 2-second twig; 3-third twig; TN-tibial nerve; CPN-common peroneal nerve; IMGA-inferior medial genicular artery; ILGA-inferior lateral genicular artery; PA-popliteal artery; PV-popliteal vein).

**Figure 5.**
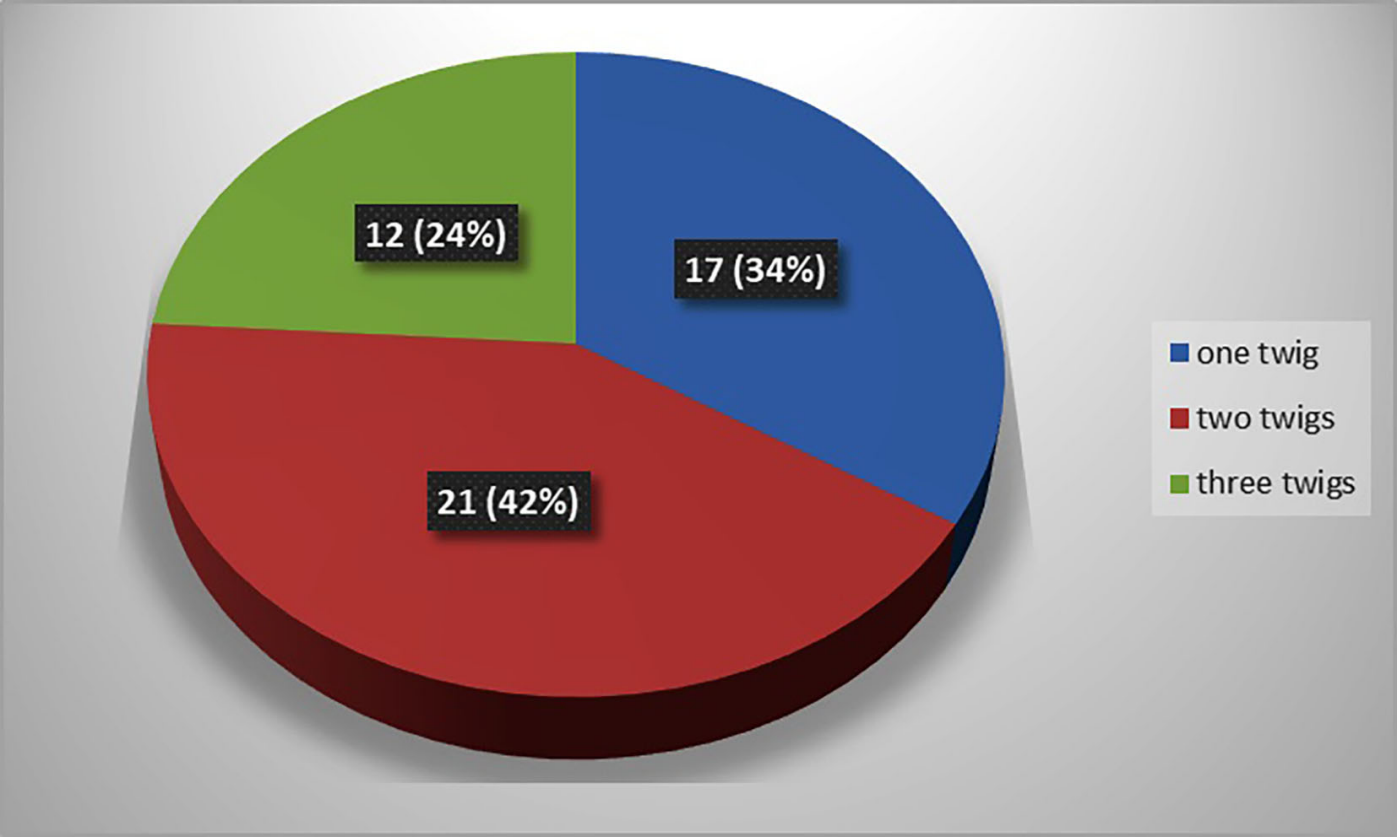
Frequency of muscular branching pattern of the nerve to popliteus (n=50).

The present study observed that, there was single twig ( Figure 2) of nerve to popliteus in 17 lower limbs (34% cases), there were two twigs ( Figure 3) in 42% cases (in 21 lower limbs) and the nerve to popliteus was giving 3 twigs ( Figure 4) in 12 lower extremities (24%). The frequency of distribution of nerve to popliteus is represented in Figure 5.

## Discussion

The popliteus muscle is the unlocking muscle of the knee and avoids the medial rotation of femur over tibia. ^7^ It is known for variations and this is explained on the basis of phylogeny. ^8^ In reptiles, fibula directly articulates with the lateral femoral condyle, so popliteus is more occupied between the proximal parts of tibia and fibula. In mammals, femur articulates with tibia, leading to the migration of popliteus muscle proximally until the lateral femoral condyle. In humans, attachment of popliteus to fibula is represented by the popliteo-fibular ligament. This is very important as it stabilizes the posterolateral aspect of the femoro-tibial articulation. According to Vani and Raveendranath, ^9^ the length and width of tendon of popliteus was 35.12 mm and 9.52 mm, which was comparable to the dimensions by Jung *et al*. ^10^ and Osti *et al*. ^11^ LaPrade *et al*. ^12^ reported that the length of popliteus tendon measures 54.5 mm. In our research, the same parameters were 24.85 ± 2.15 mm and the width at the musculotendinous junction was 8.5±1.15 mm. These dimensions are slightly lower in comparison to the data by Vani and Raveendranath. ^9^ In their study, the distance of distal attachment of popliteus from its musculotendinous junction was 107.14±13.45 mm and widest part of popliteus measured 32.38±4.33 mm. Kurtoglu *et al*. ^13^ reported in their study that popliteus muscle belly length and width were 107.14mm and 32.38mm. However, the mediolateral length of popliteus along the lower border was 89.26±14.41 mm in our study and the width of popliteus at the midpoint was 28.45±6.85 mm. These dimensions are small in comparison to Kurtoglu *et al*., ^13^ may be because of ancestral variations. Hwang *et al*. ^14^ reported the popliteal length at its lateral border, which was 119±15 mm. This was almost parallel to the findings of Vani and Raveendranath. ^9^ In the present study, this dimension was not performed and the morphometric data of the length and width of popliteus is different in our study in comparison to previous studies, as the different points were used for the measurements. The positive outcome of this anatomical research was we measured the length of the popliteus at both the upper and lower borders.

Vani and Raveendranath ^9^ reported that, the distance of origin of nerve to popliteus from the intercondylar line ranged between 12.10±10.54 mm above the intercondylar line to 18.74±11.51 mm below the intercondylar line. In the present study, this distance was measuring 27.54±6.18 mm below the intercondylar line. In most of our specimens, it was observed that, nerve to popliteus was arising separately and was not giving the nerve to soleus or nerve to tibialis posterior. We could observe that; these nerves were separate branches coming from the tibial nerve. However, previous authors mentioned that, nerve to tibialis posterior originates from the nerve to popliteus. ^14^

In the present study, it was observed that the nerve to popliteus along with the blood vessels, descend anterior to the popliteus muscle and enter at its anterior surface, which is obvious in Figure 2. The basic anatomical knowledge of these structures can enlighten the plastic surgeons during the reconstruction surgeries of popliteus. The anatomy and biomechanics of popliteus makes it an important structure, which keeps the knee stable. But its involvement is ignored in the complex injury of the knee joint. ^6^ The isolated involvement of popliteus is seen in sports injuries and it may be misinterpreted as a tear of lateral meniscus. The sports like tennis, basketball and downhill running may put additional stress on the tendon of popliteus. ^6^ The present study provided the data about the neurovascular structures in relation to the popliteus and it is believed that these details are clinically important for the effective treatment of the popliteus muscle spasticity. ^15^ Popliteus muscle tendon complex is a landmark to the operating surgeon during the sling reconstruction of popliteus tendon. ^16^ Popliteus is commonly injured in the posterolateral impact at the femoro-tibial articulation and gets torn. The muscular strains are also common in the sports injuries, which commonly affect the popliteus at its tendino-muscular junction. ^17^ Due to all these implications, the present study was undertaken. The literature search did not reveal much studies about the morphometry of the popliteus and particularly, the dataset is not available from the Indian population. In this context, the data of the present study is enlightening to the orthopedic surgeons, particularly for the posterior knee approach procedures like baker’s cyst excision, fixation of tibial plateu fractures and meniscal tears. However, the present study has limitations like the smaller sample size, and sex differences, which were not explored. In this study, repeatability of the measurements by asking a secondary observer was not performed. It would have been better if a subset of the sample was measured by a secondary observer and intraclass correlation was applied statistically. If the measurements are consistently taken by one observer incorrectly and then averaged to one value it still might not be representative of the 'true' dimensions of the vertebrae. Statistically confirming the agreement between these measurements would strengthen the quality of the research. If the intra-observer error is high, it might suggest the requirement for better measurement definitions or suggest using different tools for them in the field of vertebral morphometry in general. The correction factor for overall height of the body is not given. This is also another limitation of this study. The anatomical points for the morphometry were approximately considered, which may not be the perfect method.

## Conclusion

The present study offered the detailed morphometric data about the dimensions of the popliteus muscle belly and its tendon along with the popliteo-fibular ligament. It is believed that, the data of popliteal muscle tendon complex of this study will be enlightening to the orthopedic surgeons particularly in the field of arthroscopic and plastic surgery like the reconstruction. The data can be considered as the database for our population.

## Data Availability

Figshare: Raw Data _Popliteus.xlsx, https://doi.org/10.6084/m9.figshare.23967018.v1. ^18^ Data are available under the terms of the Creative Commons Zero “No rights reserved” data waiver (CC0 1.0 Public domain dedication).
