# Supplementary material for: Morphological study of the popliteus muscle-tendon complex in formalin embalmed adult cadavers
Source: F1000Res. 2024 Jun 5;12:1329. Originally published 2023 Oct 13. [Version 4] doi: 10.12688/f1000research.141366.4 (PMC11528185; doi:10.12688/f1000research.141366.4)
Supplement: Peer review history

# Reviewer report: Reviewer response for version 4

**DOI:** 10.5256/f1000research.167189.r330017

**Published:** 2024-10-31

**Authors:** Andrew Emmanuel Tito

**Affiliations:** 1 Mwanza University, Tanzania, Tanzania

On the Methodology

 -The author should specify how many left and right lower limbs were dissected as there have been evidences of body side-based anatomical variations of structures. This could help to deduce any side-based differences or correlation in measured parameters.

 On the Results

 -The author states that there no correlation between parameters. However I could not find any regression analysis between any variable showing a value of r=0. Despite having no observed correlation between stature and muscle/tendon length and other parameters, the author should have run a regression analysis to develop a regression equation that could predict the the length of popliteus or its tendon by using the stature as several studies have demonstrated.

 On the Discussion

 -The author mentions the reason for the observed lower values of popliteus muscle/tendon length to be **ancestral variation**, this can be expanded to provide more clarity. Also the reason for the relatively lower values could have been deduced from difference in sample sizes used between the current study and the compared ones as well as other morphometric parameters of the sample used in this study most likely the statures of cadavers

Is the work clearly and accurately presented and does it cite the current literature?

Yes

If applicable, is the statistical analysis and its interpretation appropriate?

Partly

Are all the source data underlying the results available to ensure full reproducibility?

Yes

Is the study design appropriate and is the work technically sound?

Yes

Are the conclusions drawn adequately supported by the results?

Yes

Are sufficient details of methods and analysis provided to allow replication by others?

Partly

Reviewer Expertise:

Clinical Anatomy, Anthropology, Developmental biology

I confirm that I have read this submission and believe that I have an appropriate level of expertise to confirm that it is of an acceptable scientific standard, however I have significant reservations, as outlined above.

## References

[1] : The Association between Anatomical Variants of Musculoskeletal Structures and Nerve Compressions of the Lower Limb: A Systematic Review and Meta-Analysis. *Diagnostics (Basel)* .2024;14(7) : 10.3390/diagnostics14070695 10.3390/diagnostics14070695 38611609 PMC11011940

[2] : Anatomical variations of the brachiocephalic artery and their clinical relevances: A cadaveric study of Ugandan population. *Indian Journal of Clinical Anatomy and Physiology* .2024;10(4) : 10.18231/j.ijcap.2023.056 250-256 10.18231/j.ijcap.2023.056

# Reviewer report: Reviewer response for version 4

**DOI:** 10.5256/f1000research.167189.r286864

**Published:** 2024-07-02

**Authors:** Graham Louw

**Affiliations:** 1 University of Cape Town, Cape Town, South Africa

The authors have updated the article with my suggestions. I have nothing further to add.

Is the work clearly and accurately presented and does it cite the current literature?

Yes

If applicable, is the statistical analysis and its interpretation appropriate?

Partly

Are all the source data underlying the results available to ensure full reproducibility?

Partly

Is the study design appropriate and is the work technically sound?

Partly

Are the conclusions drawn adequately supported by the results?

Partly

Are sufficient details of methods and analysis provided to allow replication by others?

Partly

Reviewer Expertise:

Medical Education; Clinical Anatomy; Embryology; Comparative Anatomy; Neurosciences; Gender-based studies in anatomical education.

I confirm that I have read this submission and believe that I have an appropriate level of expertise to confirm that it is of an acceptable scientific standard.

# Reviewer report: Reviewer response for version 3

**DOI:** 10.5256/f1000research.164610.r264543

**Published:** 2024-05-16

**Authors:** Graham Louw

**Affiliations:** 1 University of Cape Town, Cape Town, South Africa

Thank you to them for addressing some of the points raised by me.

 Regarding Figure1 and Table 1, they state the following: *The measurements of the popliteus muscle tendon complex are schematically represented in Figure 1 and tabulated in Table 1.* But what I am saying is that there is no explanation as to what anatomical structures and features have been listed as letters A through to N, as far as I can find. Whilst these may be fairly obvious to an anatomist, an author is expected to list them one by one, and be explicit.

 For some of the other points that I have raised, they basically listed them (below) as limitations of the study. I am unsure whether this is acceptable to the Journal or whether the editors require the researchers to go back and address them in a more positive / active way.

*There was no description* *of the sex, age, or statures of the cadavers that were used in this experiment. This study has failed to provide the statistical difference or correlation between these parameters. In anatomical measurements, sex differences which are related to the difference in the average stature are often observed. Especially in the lower extremities, length dramatically changes with growth. There is no correlation between stature and muscle length or any other parameters in this study. The comparison of the muscle belly thickness in the formaldehyde-fixed cadavers is one of the most challenging methods, because we might not know whether the muscle is in the contracting or relaxing phase. Moreover, the muscle belly easily changes its shape while dissecting the cadaver, even in formaldehyde-fixed cadavers.*

**

*We also did not describe the position of the knee joint. It is difficult to fixate the cadaver in a certain position because sometimes cadavers are already fixed its position by rigor mortis. These are the potential limitations of this anatomical research.*

Is the work clearly and accurately presented and does it cite the current literature?

Yes

If applicable, is the statistical analysis and its interpretation appropriate?

Partly

Are all the source data underlying the results available to ensure full reproducibility?

Partly

Is the study design appropriate and is the work technically sound?

Partly

Are the conclusions drawn adequately supported by the results?

Partly

Are sufficient details of methods and analysis provided to allow replication by others?

Partly

Reviewer Expertise:

Medical Education; Clinical Anatomy; Embryology; Comparative Anatomy; Neurosciences; Gender-based studies in anatomical education.

I confirm that I have read this submission and believe that I have an appropriate level of expertise to confirm that it is of an acceptable scientific standard, however I have significant reservations, as outlined above.

# Response

**Published:** 2024-05-28

**Authors:** B.V. Murlimanju

**Affiliations:** ANATOMY, Manipal Academy of Higher Education, Mangalore, Karnataka, India

Dear Professor, thank you very much for the comments. In this revised version, the anatomical structures and features listed as letters ‘A’ to ‘N’ are detailed and clearly explicated. They are inserted as a new table. We have checked our data collection and analysed the sex-based differences. The stature of the male and female cadavers that were used in this study and the length of the thigh are given. However, we could not provide the statistical difference or correlation between these parameters. We could not correlate between stature and muscle length. This can be considered as the limitation of this study. The position of the cadaver and the knee joint, during the dissection are given in this revised version.

# Reviewer report: Reviewer response for version 2

**DOI:** 10.5256/f1000research.160299.r238915

**Published:** 2024-02-20

**Authors:** Kyutaro Kawagishi

**Affiliations:** 1 International University of Health and Welfare, Chiba, Japan

The authors of the “Morphological study of the popliteus muscle-tendon complex in formalin embalmed adult cadavers” measured the sizes of the popliteus muscles in the 50 sides of the formaldehyde-fixed lower limbs (25 adult Indian cadavers). The authors listed data from the experiment and concluded that the results may help orthopedic surgeons.

 However, several methodological concerns arose after reading this manuscript.

 Major revision

 1. In the “Methods” section, including supplementary data, there was no description of the sex, age, or statures of the cadavers that were used in this experiment. Please describe those in the manuscript. Furthermore, please analyze whether there will be a statistical difference or correlation between parameters.

 In anatomical measurements, sex differences (which are related to the difference in the average stature) are often observed. Especially in the lower extremities, length dramatically changes with growth. The authors described that the sex differences were not explored in this study without any detailed explanation. If it is impossible to describe the sex difference, was there any correlation between stature and muscle length or any other parameters? With those analyses, this study might become more valuable.  

 2. Comparison of the muscle belly thickness in the formaldehyde-fixed cadavers is one of the most challenging methods because you might not know whether the muscle is in the contracting or relaxing phase. Moreover, the muscle belly easily changes its shape while dissecting the cadaver, even in formaldehyde-fixed cadavers.

 Furthermore, the authors did not describe the position of the knee joint. It is difficult to fixate the cadaver in a certain position because sometimes cadavers are already fixed its position by rigor mortis.

 As mentioned above, this study has a limitation. At least the authors must write those limitations in the manuscript.

Is the work clearly and accurately presented and does it cite the current literature?

Partly

If applicable, is the statistical analysis and its interpretation appropriate?

Not applicable

Are all the source data underlying the results available to ensure full reproducibility?

Partly

Is the study design appropriate and is the work technically sound?

Partly

Are the conclusions drawn adequately supported by the results?

Partly

Are sufficient details of methods and analysis provided to allow replication by others?

Partly

Reviewer Expertise:

Gross Anatomy

I confirm that I have read this submission and believe that I have an appropriate level of expertise to confirm that it is of an acceptable scientific standard, however I have significant reservations, as outlined above.

# Response

**Published:** 2024-04-04

**Authors:** B.V. Murlimanju

**Affiliations:** ANATOMY, Manipal Academy of Higher Education, Mangalore, Karnataka, India

As per the reviewer’s opinion, the potential limitations of this anatomical research are added in this revised version.

# Reviewer report: Reviewer response for version 2

**DOI:** 10.5256/f1000research.160299.r232105

**Published:** 2024-01-10

**Authors:** Graham Louw

**Affiliations:** 1 University of Cape Town, Cape Town, South Africa

Thank you for making the revisions.

 No further comments to make.

Is the work clearly and accurately presented and does it cite the current literature?

Yes

If applicable, is the statistical analysis and its interpretation appropriate?

Partly

Are all the source data underlying the results available to ensure full reproducibility?

Partly

Is the study design appropriate and is the work technically sound?

Partly

Are the conclusions drawn adequately supported by the results?

Partly

Are sufficient details of methods and analysis provided to allow replication by others?

Partly

Reviewer Expertise:

Medical Education; Clinical Anatomy; Embryology; Comparative Anatomy; Neurosciences; Gender-based studies in anatomical education.

I confirm that I have read this submission and believe that I have an appropriate level of expertise to confirm that it is of an acceptable scientific standard.

# Reviewer report: Reviewer response for version 1

**DOI:** 10.5256/f1000research.154802.r215167

**Published:** 2023-10-16

**Authors:** Graham Louw

**Affiliations:** 1 University of Cape Town, Cape Town, South Africa

Thank you for giving me the opportunity to read and comment on this submission.

 This is an interesting study and the authors have done plenty of work to ensure that the research project was covered in depth.

 I have several suggestions for improvements which I hope will prove beneficial for the authors.

 There are quite a few anatomical facts listed under the Introduction and I think that we need references for this information to be inserted.

 Under Methods, the following is stated: "Single person, who is a coauthor in this study, performed all the measurements. This was followed to prevent the inter-observer bias and the measurements were taken on three consecutive times. The average of which was considered to prevent the intra-observer bias." I do not follow this reasoning because it is far more reliable to have inter-observer participation for credible results - and I am not sure what this "bias" refers to.

 The caption for Figure 1 needs to indicate what all of those letters mean. Alternatively, the journal may be happy to have the caption refer to the details in Table 1 as the explanation.

 Under Methodology, we need to read how all of those anatomical points for the measurements were determined.

 The authors state that "The data can be considered as the database from our population." but I am unable to establish what this population is. Towards the end of their Discussion they state "The literature search did not reveal much studies about the morphometry of the popliteus and particularly, the dataset is not available from the Indian population." but the details of the population need to be given under Materials.

 The authors state that they did not explore "gender" differences but we do not know the gender identity of the bodies in the dissection hall - only their sex. the authors should state that sex differences were not explored.

 Regarding comparing left and right sides, we need to see the actual p values when deciding whether there was or was not statistical significance. Why was sidedness compared for some data and not for others?

 Did the authors consider making corrections for human variation, in other words a correction factor for overall height of the body? Or was the idea rather to report the averages of the data points used? If so, maybe the range (maximum / minimum) should be given and then the average for each one?

 For the figures illustrating the dissections, the captions need to indicate what view this is (posterior) and whether it is a left or right leg, and then provide an indication of superior / inferior / lateral / medial.

 One of the limitations is given as formalin altering the morphology of the structures - but in what way are they altered?

 This article needs to be proofread for grammar - and I am not sure who will be doing that. It is no longer the role of a reviewer to address the language used by an author. Furthermore, does the journal check all of the referencing?

Is the work clearly and accurately presented and does it cite the current literature?

Yes

If applicable, is the statistical analysis and its interpretation appropriate?

Partly

Are all the source data underlying the results available to ensure full reproducibility?

Partly

Is the study design appropriate and is the work technically sound?

Partly

Are the conclusions drawn adequately supported by the results?

Partly

Are sufficient details of methods and analysis provided to allow replication by others?

Partly

Reviewer Expertise:

Medical Education; Clinical Anatomy; Embryology; Comparative Anatomy; Neurosciences; Gender-based studies in anatomical education.

I confirm that I have read this submission and believe that I have an appropriate level of expertise to confirm that it is of an acceptable scientific standard, however I have significant reservations, as outlined above.

# Response

**Published:** 2023-12-16

**Authors:** B.V. Murlimanju

**Affiliations:** ANATOMY, Manipal Academy of Higher Education, Mangalore, Karnataka, India

**1. Reviewer comment: **This is an interesting study and the authors have done plenty of work to ensure that the research project was covered in depth.

**Author Reply: **We thank the reviewer for the kind words and appreciating our research.

**2. Reviewer comment: **There are quite a few anatomical facts listed under the Introduction and I think that we need references for this information to be inserted.

**Author Reply: **We accept the reviewer opinion. As per the suggestion, citations are added for this literature. Three more references were added in the introduction section.

**3. Reviewer comment:** Under Methods, the following is stated: "Single person, who is a coauthor in this study, performed all the measurements. This was followed to prevent the inter-observer bias and the measurements were taken on three consecutive times. The average of which was considered to prevent the intra-observer bias." I do not follow this reasoning because it is far more reliable to have inter-observer participation for credible results - and I am not sure what this "bias" refers to.

**Author Reply:** In this study, repeatability of the measurements by asking a secondary observer was not performed. It would have been better if a subset of the sample was measured by a secondary observer and intraclass correlation was applied statistically. If the measurements are consistently taken by one observer incorrectly and then averaged to one value it still might not be representative of the 'true' dimensions of the vertebrae. Statistically confirming the agreement between these measurements would strengthen the quality of the research. If the intra-observer error is high, it might suggest the requirement for better measurement definitions or suggest using different tools for them in the field of vertebral morphometry in general.

 This information is added as a limitation of this study.

**4. Reviewer comment:** The caption for Figure 1 needs to indicate what all of those letters mean. Alternatively, the journal may be happy to have the caption refer to the details in Table 1 as the explanation.

**Author Reply:** As per the reviewer suggestion, alternative option, refer to the details in Table 1 is added in the revised version.

**5. Reviewer comment:** Under Methodology, we need to read how all of those anatomical points for the measurements were determined.

**Author Reply:** These anatomical points were approximately considered, which may not be the perfect morphometric assessment. This can be considered as another limitation of this manuscript and is added as a limitation in the revised version.

 .

 6. **Reviewer comment:** The authors state that "The data can be considered as the database from our population." but I am unable to establish what this population is. Towards the end of their Discussion they state "The literature search did not reveal much studies about the morphometry of the popliteus and particularly, the dataset is not available from the Indian population." but the details of the population need to be given under Materials.

**Author Reply:** As per the reviewer opinion, the details of the population is now given under the materials and methods.

**7. Reviewer comment:** The authors state that they did not explore "gender" differences but we do not know the gender identity of the bodies in the dissection hall - only their sex. the authors should state that sex differences were not explored.

**Author Reply:** As per the opinion, it is now mentioned that the sex differences were not explored.

**8. Reviewer comment:** Regarding comparing left and right sides, we need to see the actual p values when deciding whether there was or was not statistical significance. Why was sidedness compared for some data and not for others?

**Author Reply:** The study measured 17 parameters and all were compared over the right and left sides. They are given in tables 6, 7 and 8. Individual ‘p’ values are given in the raw data.

**9. Reviewer comment:** Regarding comparing left and right sides, we need to see the actual p values when deciding whether there was or was not statistical significance. Why was sidedness compared for some data and not for others?

**Author Reply:** The study measured 17 parameters and all were compared over the right and left sides. They are given in tables 6, 7 and 8. Individual ‘p’ values are given in the raw data.

**10. Reviewer comment: **Did the authors consider making corrections for human variation, in other words a correction factor for overall height of the body? Or was the idea rather to report the averages of the data points used? If so, maybe the range (maximum / minimum) should be given and then the average for each one?

**Author Reply:** The correction factor for overall height of the body is not given, which is a limitation of this study. Since we applied the paired ‘t’ test, the mean and standard deviation were considered instead of range and average.

**11. Reviewer comment: **For the figures illustrating the dissections, the captions need to indicate what view this is (posterior) and whether it is a left or right leg, and then provide an indication of superior / inferior / lateral / medial.

**Author Reply: **As per the reviewer opinion, figures and figure legends were revised. The details of the view, side and the position were added.

**12. Reviewer comment: **For the figures illustrating the dissections, the captions need to indicate what view this is (posterior) and whether it is a left or right leg, and then provide an indication of superior / inferior / lateral / medial.

**Author Reply: **As per the reviewer opinion, figures and figure legends were revised. The details of the view, side and the position were added.

**13. Reviewer comment: **One of the limitations is given as formalin altering the morphology of the structures - but in what way are they altered?

**Author Reply: **We did not get the explanation for our writing. So, this limitation is removed in this revised version. We accept the reviewer opinion.

**14. Reviewer comment: **This article needs to be proofread for grammar –

**Author Reply: **In this revised version, the manuscript was revised with respect to the written English. We utilized the online software www.gramarly.com.

 B.V. Murlimanju, MD

 Corresponding Author
